# Treadmill exercise prevents recognition memory impairment in VD rat model and enhancement of hippocampal structural synaptic plasticity

**DOI:** 10.1002/brb3.3633

**Published:** 2024-07-25

**Authors:** Linlin Zhang, Hao Wu, Yongzhao Fan, Fang Tian

**Affiliations:** ^1^ Department of physical education Henan normal university Xinxiang China; ^2^ Beijing Key Laboratory of Sports Function Assessment and Technical Analysis Capital University of Physical Education and Sports Beijing China; ^3^ Department of Physical Education Nanjing Medical University Nanjing China

**Keywords:** recognition memory, structural synaptic plasticity, treadmill exercise, VD

## Abstract

**Objective:**

In vascular dementia (VD), memory impairment caused by the damage of synaptic plasticity is the most prominent feature that afflicts patients and their families. Treadmill exercise has proven beneficial for memory by enhancing synaptic plasticity in animal models including stroke, dementia, and mental disorders. The aim of this study was to examine the effects of treadmill exercise on recognition memory and structural synaptic plasticity in VD rat model.

**Methods:**

Male Sprague‐Dawley rats were randomly assigned into four groups: control group (C group, *n* = 6), vascular dementia group (VD group, *n* = 6), treadmill exercise and vascular dementia group (Exe‐VD group, *n* = 6), and treadmill exercise group (Exe group, *n* = 6). Four‐week treadmill exercise was performed in the Exe‐VD and Exe groups. Then, the common carotid arteries of rats in the VD and Exe‐VD groups were identified to establish the VD model. Behavior tests (open‐field test and novel recognition memory test) were adopted to evaluate anxiety‐like behavior and recognition memory. Transmission electron microscopy and Golgi staining were performed to observe synaptic ultrastructure and spine density in the hippocampus.

**Results:**

Our study demonstrated that VD rat exhibited significantly anxiety‐like behavior and recognition impairment (*p* < .01), while treadmill exercise significantly alleviated anxiety‐like behavior and improved recognition memory in VD rat (*p* < .01). Transmission electron microscopy revealed that hippocampal synapse numbers were significantly decreased in the VD group compared to the control group (*p* < .05). These alterations were reversed by treadmill exercise, and the rats exhibited healthier synaptic ultrastructure, including significantly increased synapse (*p* < .05). Meanwhile, golgi staining revealed that the spine numbers of the hippocampus were significantly decreased in the VD group compared to the control group (*p* < .05). When compared with the VD group, hippocampal spine numbers were significantly increased in the Exe‐VD group (*p* < .05).

**Conclusion:**

The improvement of VD‐associated recognition memory by treadmill exercises is associated with enhanced structural synaptic plasticity in VD rat model.

## Graphical abstract

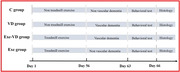

## 1 INTRODUCTION

Vascular dementia (VD) is considered to be the second most common form of dementia after Alzheimer's disease (AD) and accounts for at least 20% of dementia cases (Akhter et al., 2021; Santiago‐Mujika et al., 2021). VD may be caused by cerebrovascular disease, including ischemic or hemorrhagic stroke, and hypoperfusion ischemic brain injury due to cardiovascular and circulatory disorders (Hachinski et al., 2019). The persistent and irreversible memory impairment in VD patients leads to a serious deterioration in the quality of life and places a heavy economic burden on the families of patients (Livingston et al., 2020; Y. M. Zhang et al., 2017). Together with the increasing age of the population and improved survival rates from cardiovascular diseases, VD may affect more individuals in the future (T O'Brien & Thomas, 2015). Therefore, the prevention and treatment of VD is increasingly important at home and abroad, especially in countries with aging populations. It was mentioned as early as the 1980s that VD at present may be more amenable to prevention and treatment than AD (Skoog, 1994). At present, many drugs may prevent the degradation of memory in VD patients, including donepezil and tanzhi granules, which improve cognitive impairment by inhibiting neuroinflammation and acetylcholinesterase activity (Linh et al., 2022). However, adverse effects of such medicines include dependence and depression.

As a non‐pharmacological treatment, physical exercise has proven beneficial in bolstering brain health and function by reducing brain inflammation (de Miguel et al., 2021), neuroinflammation (Mee‐Inta et al., 2019), and redistributing blood flow and neural activity (Thomas et al., 2020). Among new therapeutic strategies being pursued to minimize cognitive damage, clinical studies have confirmed that physical exercise is associated with lower incidence of vascular dementia (Hansson et al., 2019). Moreover, various animal models have demonstrated that regular volunteer running or treadmill exercise can improve memory function in VD rats, in association with the protection of astrocyte function (Cao et al., 2022; Leardini‐Tristão et al., 2020). However, the relationship between structural synaptic plasticity and the mechanism by which exercise improves memory still needs to be further explored. The brain changes throughout life at synaptic levels, including morphological and physiological changes. Structural synaptic plasticity is relative to synaptic morphology, which is thought to underlie higher cognitive processes such as memory storage and recall (Andrade‐Talavera & Rodríguez‐Moreno, 2021). The role of structural synaptic plasticity between physical exercise and memory function has been recognized on both animal models and humans. Strong clinical and experimental evidence support that during exercise, modifications in the morphology of synaptic generate structural synaptic plasticity changes that are assumed to underlie enhanced cognitive processes such as memory function (Herold et al., 2019; Thomas et al., 2020). Furthermore, in vivo evidence has been provided to illustrate the molecular mechanisms of exercise on memory function possibly via regulating dendritic spine formation (Ivy et al., 2020) and the ultrastructural morphology of synapses (Fattoretti et al., 2018), in addition to memory recovery in VD (L. Zhang et al., 2020) and other mental diseases (Miller et al., 2018). These findings suggest an important role for structural synaptic plasticity in the pathogenesis of VD, and it has become an interesting target for therapeutic intervention. However, the relationship between structural synaptic plasticity and the mechanism by which exercise improves memory still needs to be further explored. Here, the aim of our present study is to determine the regulatory paradigm of physical exercise on memory function and structural synaptic plasticity in the hippocampus under VD model.

## 2 METHODS

### 2.1 Experimental animals and grouping

**FIGURE 1:**
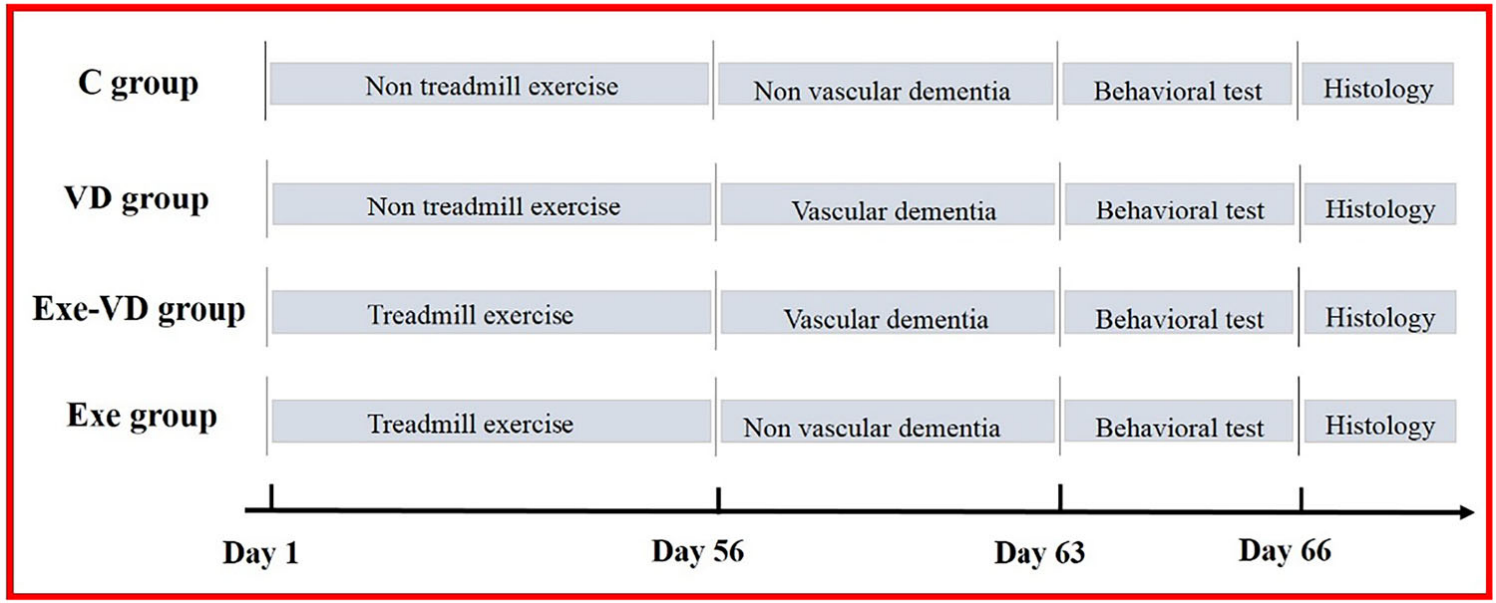
The experimental timeline. C group, control group; Exe group, treadmill exercise group; Exe‐VD group, treadmill exercise and vascular dementia group; VD group, vascular dementia group.

Male Sprague‐Dawley rats (8 weeks of age) were purchased from Shanghai SLAC Laboratory Animal Co., Ltd and housed three per cage under normal light–dark cycle with food and water (temperature: 22 ± 3°C; humidity: 40%–70%). All rats were randomly assigned into four groups (*n* = 6 each): control group (C group), vascular dementia group (VD group), treadmill exercise and vascular dementia group (Exe‐VD group), and treadmill exercise group (Exe group). Body weight was measured every 3 days, and other treatments were performed at designated times according to the experimental timeline (Figure 1). All the animal procedures and methods conducted in this study were approved by the Ethics Committee of Experimental Animals of Capital University of Physical Education and Sport (Approval number 2020A76).

### 2.2 Treadmill exercise

The treadmill exercise was performed according to previous literature (L. Zhang et al., 2022). During the adaptation period, the exercised rats were allocated in the test room for 30 min acclimation, followed by treadmill running for 30 min on 3 consecutive days (first day at 8 m/min, second day at 10 m/min, and third day at 12 m/min). Then, those rats were subjected to a treadmill exercise protocol at the speed of 12 m/min for 60 min. In total, rats were trained for 1 h per day, 5 days per week, for a total of 4 weeks.

### 2.3 VD model

The VD model was induced by permanent bilateral common carotid artery stenosis, according to a previously reported operation (G. Zhang et al., 2008). First, under deep anesthesia with 10% chloral hydrate (350 mg/kg), the common carotid arteries of rats in VD and Exe‐VD groups were identified and isolated from vagus nerves bilaterally through a ventral midline cervical incision, then tightly ligated using 10‐0 suture thread on both ends. The rats in the control group were subjected to the same surgical procedure except that the common carotid arteries were exposed but not ligated. The “Zea‐Longa” five‐point scale (Table 1) was performed according to previously described methods to verify whether the animal model had been successfully established. A score between 1 and 4 indicates successful modeling of VD.

**TABLE 1:** “Zea‐Longa” five‐point scale.

Point	Symptom
Zero point	No neurological deficit
One point	Failure to extend left forepaw fully
Two points	Circling to the left
Three points	Falling to the left
Four points	No spontaneous walking with depressed level of consciousness
Five points	Death

### 2.4 Behavior test

Behavior examination included the open‐field test and recognition memory test. On the first day, the experimental apparatus of the open‐field test was a black open‐field box (100 cm × 100 cm × 50 cm) positioned in a dimly illuminated room. Each rat was allowed to explore this environment for 10 min freely. During the test session, a computer‐based system automatically measured the moving distance, time in the center area, and number of times to enter the center area (Pang et al., 2022). On the second day, each rat was placed in the black open‐field box containing two identical objects and allowed to explore two identical objects (A1 and A2) for 10 min. The object with most object interactions were defined as a similar object. After 5 min or 24 h, the similar object was replaced with a novel object, and the rats were allowed to explore for 10 min. A discrimination index was calculated as the difference in number exploring the novel and familiar object, expressed as the ratio of the total number spent exploring both objects (i.e., [Number Novel/Number Novel + Number Familiar] × 100%). In addition, all object combinations and locations were used in a balanced manner to reduce potential bias due to preferences for specific locations or objects (Y.‐F. Chen et al., 2022).

### 2.5 Transmission electron microscopy

After behavior test, all the rats were anesthetized with chloral hydrate (350 mg/kg, i.p.), and the hippocampus brain region was collected on ice plate. Transmission electron microscopy was performed to observe synaptic ultrastructure in the hippocampus (Kubota et al., 2018). The hippocampal rat brain tissue was cut within 1–2 mm^3^ and blocked with fresh transmission electron microscopy fixative for 2 h. Then, the tissues were washed using 0.1 M phosphate buffered saline (pH 7.4) five times and fixed with 1% OsO4 in 0.1 M PB (pH 7.4) for 7 h at room temperature. After washing with phosphate buffered saline, the tissues were dehydrated in graded ethanol solutions (30%–100%). Brain tissues were stained with resin penetration (acetone: EMBed 812 = 3:1 for 2–4 h, acetone: EMBed 812 = 1:1 overnight, acetone: EMBed 812 = 1:3 for 2–4 h, pure EMBed 812 for 5–8 h) at an oven set at 37°C overnight. Next day, the tissues were moved into an oven set at 65°C to polymerize for 2 days. Subsequently, the tissues were cut to 60–80 nm thin and fished out onto the 150 meshes cuprum grids with formvar film. Finally, the brain tissue sections are placed into 2% uranium acetate saturated alcohol solution and 2.6% lead citrate staining for 8 min, for drying overnight at room temperature. Images were captured with a transmission electron microscope (Hitachi).

### 2.6 Golgi staining

Golgi staining was performed as previously described. Briefly, the fresh whole brain was obtained and immediately fixed with 4% paraformaldehyde for 48 h. Then, the sample was placed in to a 45‐mL EP tube containing Golgi‐cox staining solution for 14 days. After treatment with distilled water, 80% glacial acetic acid and 30% sucrose, respectively, tissues were cut into 100 microns and dried in the dark overnight. The sections were slides with concentrated ammonia water and hardening fixing solution for 15 min, then washing with distilled water for 3 min, dry, and seal the section with glycerin gelatin. Images of the brain tissue by panoramic scanning with digital slice scanner (Vints et al., 2019).

### 2.7 Statistics

Data in this experiment are presented as the mean ± SEM. Data sets were compared with two‐way analysis of variance (ANOVA) followed by Tukey's post hoc analysis. Post hoc analyses were performed only when ANOVA yielded a significant main effect or a significant interaction between the two factors. Results were considered to be significant at *p* < .05.

## 3 RESULTS

### 3.1 Neurobehavioral assessment of VD model

**FIGURE 2:**
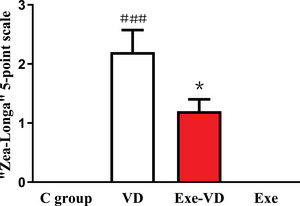
Neurological defect score in each group. ^###^ *p* < .001 versus C group; ^*^ *p* < .05 versus VD group. C group, control group; Exe group, treadmill exercise group; Exe‐VD group, treadmill exercise and vascular dementia group; VD group, vascular dementia group.

A neurological evaluation was performed 7 days after 2‐VO, and scores were recorded on a five‐point scale according to Table 1. As shown in Figure 2, rats in the control group had no neurological deficit. The results also showed that the grade score was significantly higher in the VD group when compared with the C group (*p* < .001; Figure 2). In addition, compared with the VD group, the rats in the Exe‐VD group significantly had the low‐grade score (*p* < .05; Figure 2). Thus, we concluded that vascular dementia was successfully developed in our experiment undergoing the 2‐VO procedure.

### 3.2 VD‐induced anxiety behavior was ameliorated by treadmill exercise

**FIGURE 3:**
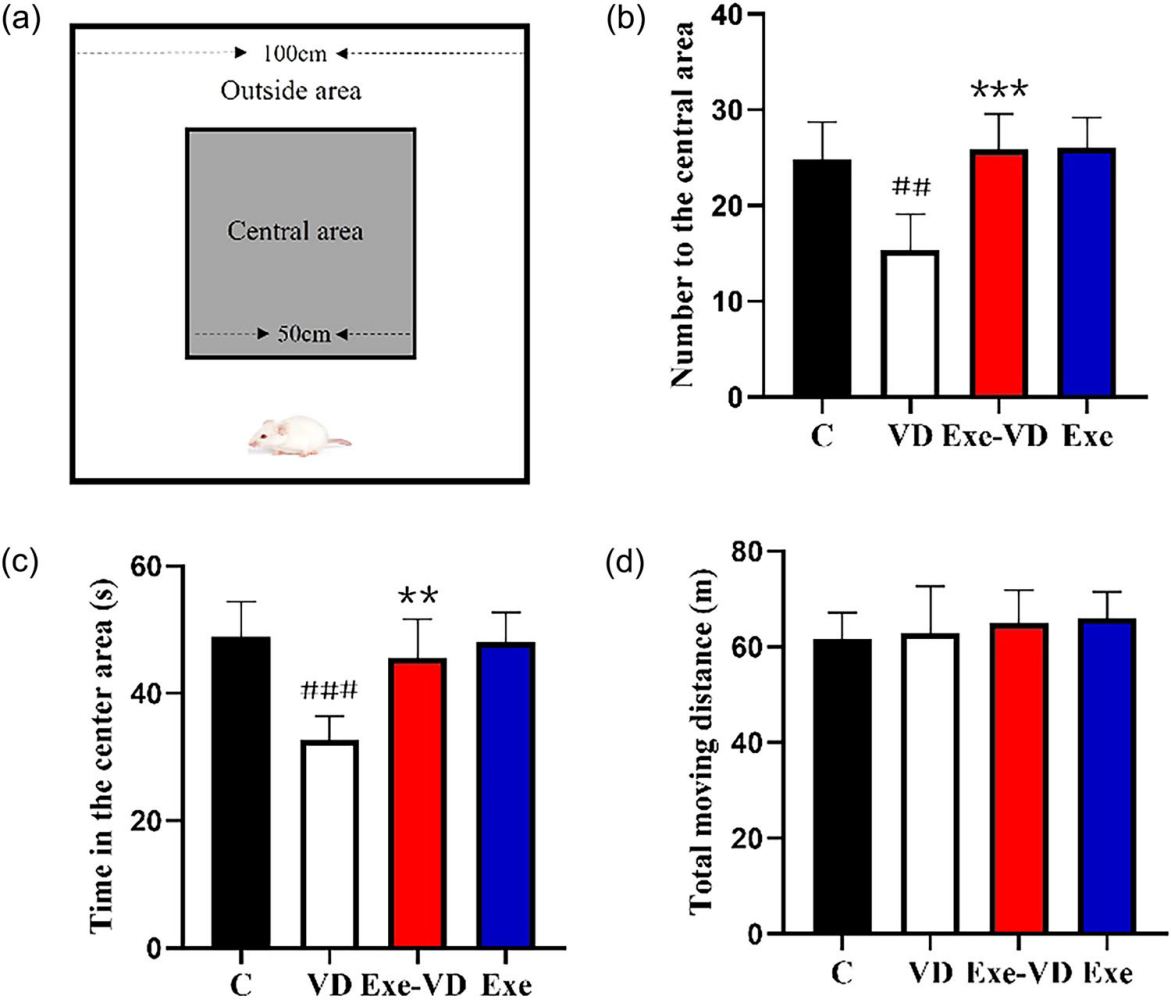
Treadmill exercise prevented stress‐induced anxiety‐like behavior in the open‐field test. The figure shows (a) schematic diagram of the open‐field test, (b) number to the central area, (c) time in the center area, and (d) total moving distance. ^##^ *p* < .01 and ^###^ *p* < .001 versus C group; ^**^ *p* < .01 and ^***^ *p* < .05 versus VD group. C group, control group; Exe group, treadmill exercise group; Exe‐VD group, treadmill exercise and vascular dementia group; VD group, vascular dementia group.

In the open‐field test, the VD rats had fewer number of times to enter the central area and spent less time in the center area than control rats (*p* < .01; Figure 3b–c), whereas the total distances traveled within the open field were the same between the control and VD groups (*p* > .05; Figure 3d). After exercise intervention, the Exe‐VD cohort had a significantly elevated number of times to enter the central area and time in the center area compared to the VD group (*p* < .01, *p* < .001; Figure 3b–c), whereas the total moving distance does not differ between the VD and Exe‐VD groups (*p* > .05; Figure 3d). This result suggests that the VD rats have a mild anxiety, while their locomotor activity is normal. In addition, the anxiety behavior in VD rats can be rescued by physical exercise.

### 3.3 VD‐induced recognition memory impairment was ameliorated by treadmill exercise

**FIGURE 4:**
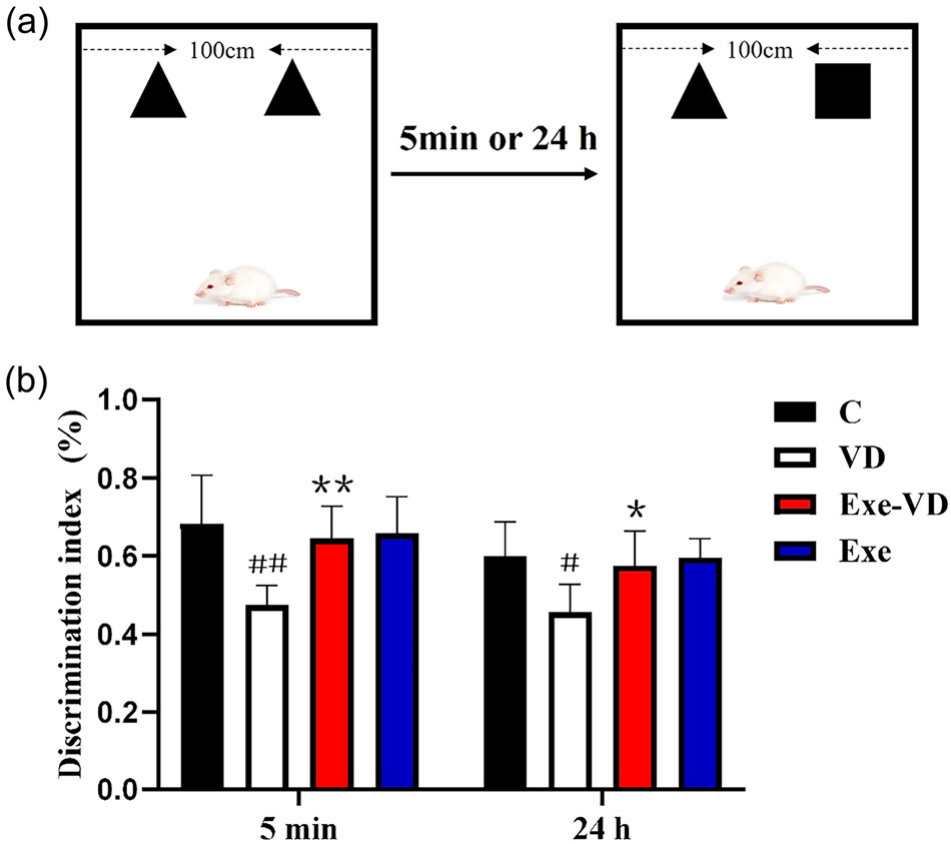
Treadmill exercise prevents recognition memory impairment in vascular dementia (VD) rats. The figure shows (a) schematic diagram of novel object recognition test, (b) the discrimination index of each group after 5 min and 24 h. ^#^ *p* < .05 and ^##^ *p* < .01 versus C group; ^*^ *p* < .05 and ^**^ *p* < .01 versus VD group. C group, control group; Exe group, treadmill exercise group; Exe‐VD group, treadmill exercise and vascular dementia group; VD group, vascular dementia group.

As shown in Figure 4a, the discrimination index of each group was obtained by a recognition memory test. When compared with control rats, the discrimination index of VD rats was significantly lesser (*p* < .05, *p* < .01; Figure 4b). Conversely, the discrimination index of Exe‐VD rats was significantly greater than VD rats (*p* < .05, *p* < .01; Figure 4b). Thus, these findings indicate that treadmill exercise rescued recognition memory impairment in the VD model.

### 3.4 Treadmill exercise rescued VD‐induced synaptic ultrastructure deficits

**FIGURE 5:**
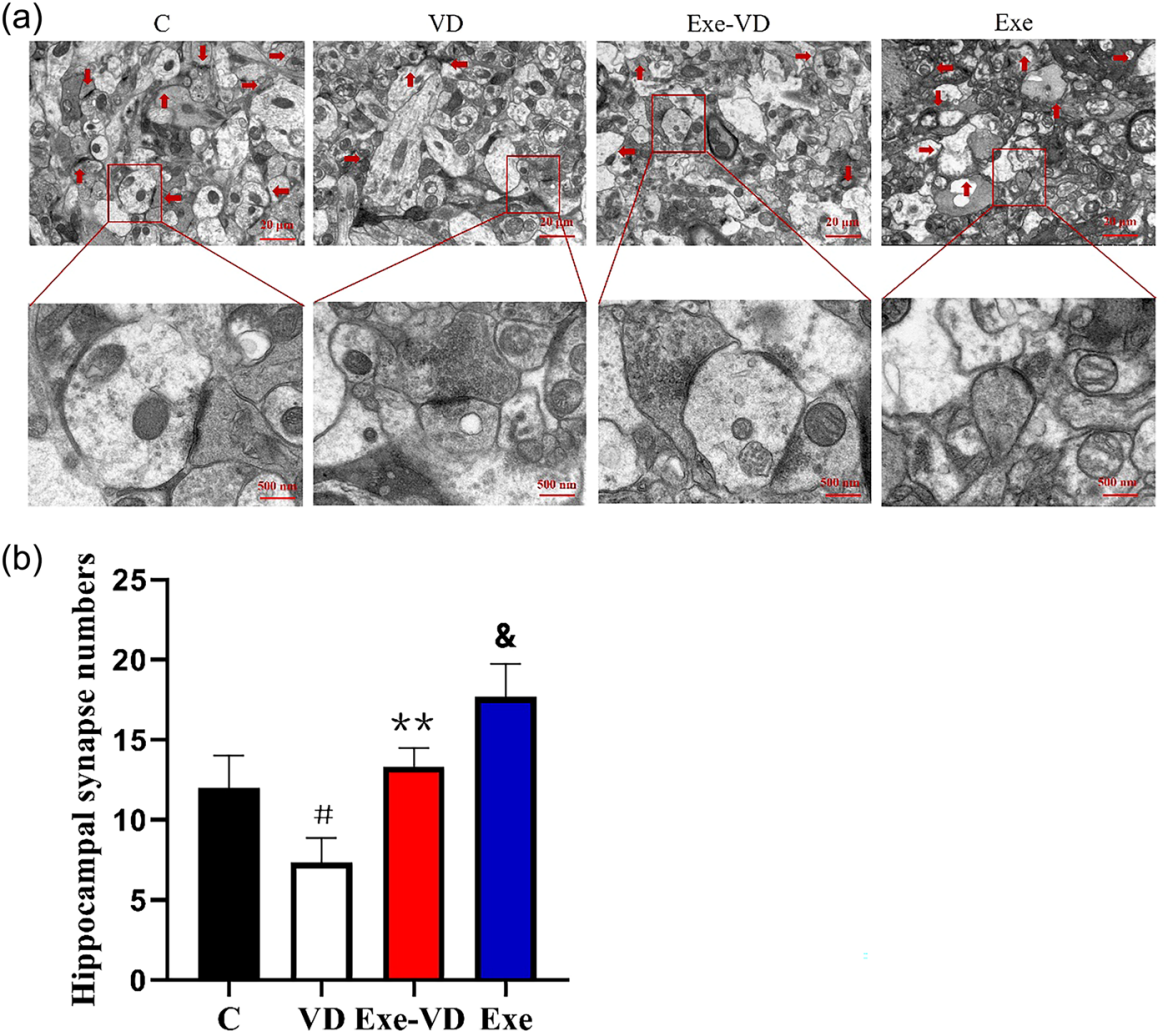
Treadmill exercise increases synapse numbers of hippocampus in vascular dementia (VD) rats. (a) Representative electron microscope images in each group. The synapses are marked by the red arrowheads. (b) The number of hippocampal synapse in each group. ^#^ *p* < .05 and ^&^ *p* < .05 versus C group; ^**^ *p* < .01 versus VD group. C group, control group; Exe group, treadmill exercise group; Exe‐VD group, treadmill exercise and vascular dementia group; VD group, vascular dementia group.

Synaptic ultrastructure was the basis of structural synaptic plasticity. Thus, we assessed hippocampal synapse numbers by transmission electron microscope that are critical for the transmission of information related to learning and memory. As shown in Figure 5a, hippocampal synapse numbers were significantly decreased in the VD group compared to the control group (*p* < .05; Figure 5b). These alterations were reversed by treadmill exercise, and the rats exhibited healthier synaptic ultrastructure, including significantly increased synapse (*p* < .01; Figure 5b). Meanwhile, in the Exe group, the number of synapses in the hippocampus was significantly increased than those in the C group (*p* < .05; Figure 5b). In conclusion, treadmill exercise ameliorated the above damage and protected the ultrastructure of synapses, suggesting improved structural synaptic plasticity.

### 3.5 Treadmill exercise rescued dendritic damage of synapse caused by VD

**FIGURE 6:**
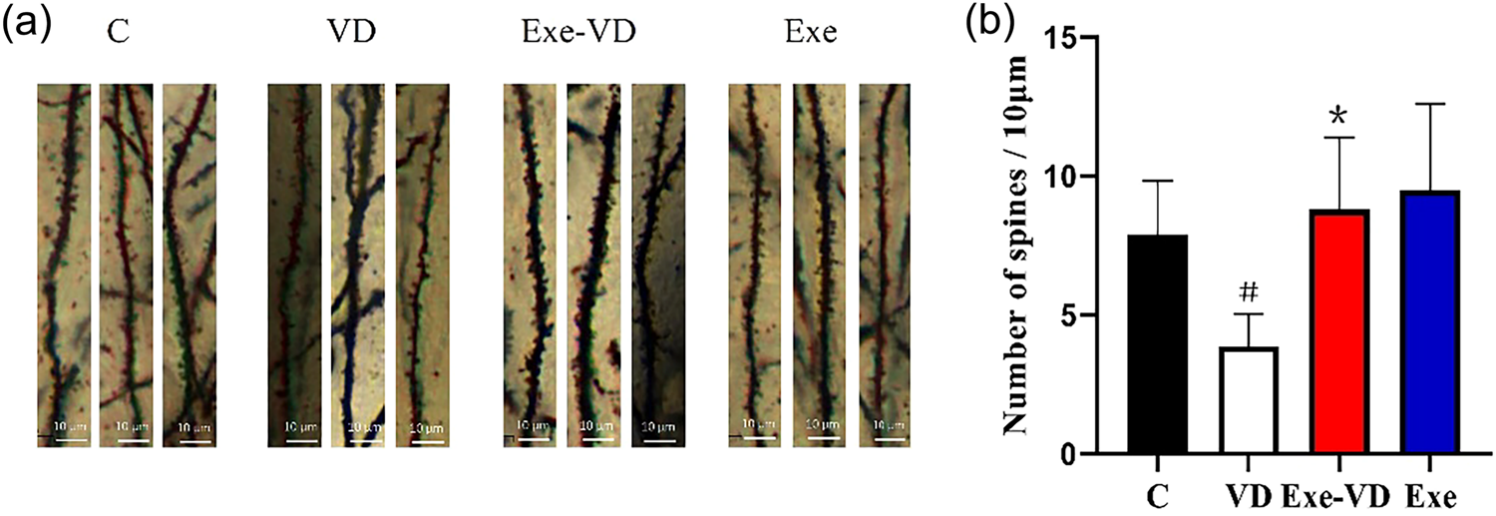
Exercise rescued spine density caused by vascular dementia (VD). (a) Representative hippocampus microphotographs of the spines on secondary dendrites of neurons in each group. (b) Number of spines per 10 µm in each group. ^#^ *p* < .05 versus C group; ^*^ *p* < .05 versus VD group. C group, control group; Exe group, treadmill exercise group; Exe‐VD group, treadmill exercise and vascular dementia group; VD group, vascular dementia group.

To further confirm if the neuroprotective effect of treadmill exercise on recognition in VD rats was associated with structural synaptic plasticity, we next analyzed spine density (Figure 6a). The results demonstrated that the spine numbers of the hippocampus were significantly decreased in the VD group compared to the control group (*p* < .05; Figure 6b). Meanwhile, compared with the VD group, hippocampal spine numbers were significantly increased in the Exe‐VD group (*p* < .05; Figure 6b). Thus, treadmill exercise blocked a decrease in the spine density of hippocampus in VD rats.

## 4 DISCUSSION

Our study aimed at understanding the link between exercise and AD have focused on structural synaptic plasticity. In this study, we used recognition memory testing coupled with transmission electron microscopy and Golgi staining visualization to track the dynamics of structural synaptic plasticity in the hippocampus after treadmill exercise on VD rats. We found that exercise promotes the restoration of synaptic ultrastructure and spine density in the hippocampus, in addition to improving recognition memory in the VD model. Our findings suggest that strengthening structural synaptic plasticity may represent a potential mechanism by which treadmill exercise prevents impairment of recognition memory in the VD model.

In our study, we first found that the discrimination index in VD rats was significantly lesser than control rats when exposed to the novel object 5 min and 24 h after they were familiarized with an identical set of objects. As the disease progresses, patients experience progressive memory loss and emotional disorder in daily life, including communication disorder and anxiety (Ballard et al., 2000; Iadecola, 2013). In vivo study also confirms impaired discrimination index in recognition memory test (Khodir et al., 2022), which is one of the most common paradigms to assess hippocampal‐dependent memory, including short‐term recognition memory and long‐term recognition memory (Sakaguchi & Sakurai, 2020). In general, short‐term memories only refer to the short‐term storage of information, whereas long‐term memories are required for remembering information (Norris, 2017). In addition, we also provided in vivo evidence that VD rats have mild anxiety in the open‐field test, a finding consistent with clinical investigation. Thus, we demonstrated the impairment of recognition memory and anxiety‐like behavior in VD progression. Furthermore, our study also revealed that treadmill exercise rescued recognition memory impairment and anxiety‐like behavior in VD model in the recognition memory test. This result was consistent with previous studies (Ohtomo et al., 2021; Park et al., 2020). Taken together, VD rats exhibited impairment in recognition memory, and the robust decrease of errors under testing indicates that treadmill exercise pretreatments prevent decline in recognition memory in the VD rat model.

Next, we examined the potential mechanisms that might underline the treadmill exercise‐induced improvement of recognition memory function in VD rats. It has been suggested that hippocampal structural synaptic plasticity constitutes the cellular basis of learning and memory, which requires the connections of synapses (Mu et al., 2022). Our results showed decreased synapse numbers in the hippocamps of VD rats. In normal state, presynaptic terminal secretes memory‐related substances via a canonical release machinery, while postsynaptic specialization senses substances via diverse receptors (Südhof, 2018). Therefore, the changes of synapse of synapse number are bound to affect synaptic structural plasticity. The study performed by Huang et al. (2021) on a murine model of vascular dementia by HE staining further indicates significant neuronal damage in the hippocampal of VD rat. Khan et al. (2024) further demonstrated that permanent bilateral common carotid artery stenosis caused changes in neuronal morphology and cell death in the cortex and hippocampus. In a neural circuit, synapses rapidly transport information between neurons while also transforming it (Südhof, 2021). In this study, we also found that treadmill exercise led to an increase in the hippocampal synapse numbers of VD rats. Meanwhile, treadmill exercise increased the synapse numbers in the hippocampus in the control group. The form and rearrangement of synapses under exercise is associated with the enhancement of structural synaptic plasticity. Animal studies have demonstrated exercise‐induced enhancement of structural synaptic plasticity by the regulation of synaptic formation and rearrangement into normal states (Chatzi et al., 2019; He et al., 2018). Thus, our study sheds light on the increase of synapse number by exercise that could be effective in exerting beneficial effects in VD rats. Axons, dendrites, and dendritic spines constitute the structural basis of synaptic plasticity. The axon is functionally specialized to transmit signals, whereas the dendrites are specialized to receive signals (Mikhaylova et al., 2020). In vivo imaging by Fluorojade B (FJB) staining revealed impairment of axonal and dendritic in the hippocampus after vascular dementia (Jiang et al., 2021). Dendritic spines are specialized postsynaptic structures that transduce presynaptic signals, are regulated by neural activity, and are correlated with learning and memory (Tazerart et al., 2020). Our findings supported that the spine numbers of hippocampus were significantly decreased in the VD group compared to the control group. A review by Frankfurt and Luine (2015) reported that a strong relationship between dendritic spine in the hippocampus and memory has been demonstrated in different spatial memory tests. Our study further revealed that treadmill exercise increased the spine numbers of hippocampus in VD rats. It is likely that treadmill exercise pretreatment potentiates synaptic connections via an increase in dendritic spines under normal and dementia conditions (K. Chen et al., 2019; Mu et al., 2022). Such mechanisms might explain why treadmill exercise ameliorates the impairment of recognition memory in the VD rat model.

In summary, treadmill exercise improved recognition memory, which can be contributed to the enhancement of hippocampal synapses number and dendritic spine density in VD rats. Our results collectively establish the central role of structural synaptic plasticity for neural network adaptations to exercises and provide more evidence for clinical intervention of memory deficits using exercise interventions.

## 5 CONCLUSIONS

Strengthening structural synaptic plasticity may represent a potential mechanism by which treadmill exercise prevents decline in recognition memory and synapse loss in 2‐VO‐induced VD rat model.

## AUTHOR CONTRIBUTIONS

**Linlin Zhang**: Conceptualization; formal analysis; investigation; writing—original draft. **Hao Wu**: investigation; methodology. **Yongzhao Fan**: Data curation; methodology. **Fang Tian**: Resources; writing—review and editing.

## CONFLICT OF INTEREST STATEMENT

The authors declare no conflicts of interest.

### PEER REVIEW

The peer review history for this article is available at https://publons.com/publon/10.1002/brb3.3633.

## Data Availability

The data sets are available from the first author on reasonable request.
